# Frequency and clinical characteristics of nitrous oxide use disorder among French health professions students

**DOI:** 10.1192/j.eurpsy.2023.346

**Published:** 2023-07-19

**Authors:** A. Dervaux, A. Szusterman, B. Angerville, L. Blecha, A. Benyamina, M. Naassila

**Affiliations:** ^1^Service d’Addictologie, EPS Barthélémy Durand, Etampes; ^2^Service d’Addictologie, Centre Hospitalier Paul Brousse, Villejuif; ^3^Service d’Addictologie, EPS Barthelémy Durand, Etampes; ^4^Service d’Addictologie, Centre Hospitalier Paul Brousse, Villejuif; ^5^Unité Inserm 1247 Groupe de Recherche sur l’Alcool et les Pharmacodépendances, Université de Picardie Jules Verne, Amiens, France

## Abstract

**Introduction:**

Nitrous oxide recreational use and abuse has significantly increased in recent years among youth. To our knowledge, no previous study investigated the frequency and characteristics of N2O use disorder.

**Objectives:**

To assess the frequency and the socio-demographic and clinical characteristics of nitrous oxide use disorder in a sample of health professions students.

**Methods:**

An online survey was distributed to health professions students at Paris-Cité University, Paris-Sorbonne University, Paris-Saclay University, Lille University and Picardy University, France. The following data were collected: age, gender, frequency of nitrous oxid use, DSM-5 criteria for nitrous oxide use disorder, frequency (day/week/month/year/lifetime) of tobacco, alcohol, cannabis, cocaine, amphetamines, and hallucinogens use, Alcohol Use Disorders Identification Test scores (AUDIT), the Fagerström Tobacco Dependence Test scores, the Cannabis Abuse Screening Test scores, lifetime psychiatric or sleep disorders.

**Results:**

2067 participants (mean age 21.7±2.6 years, 75% female) completed the survey from September 2021 to May 2022. Among them, 38% (n=790) reported nitrous oxide lifetime use. Seven per cent of the subjects (n=137) fulfilled DSM-5 criteria for current nitrous oxide use disorder (114 mild use disorder, 16 moderate use disorder, 7 severe use disorder). In the group of patients with nitrous oxide use disorder, there were correlations between nitrous oxide use disorder and daily alcohol use (Chi2=24.2, p<0.0001), daily tobacco use (Chi2=25.3, p<0.0001), AUDIT scores >12 (Chi2=7.9, p<0.0001), lifetime depressive disorders (Chi2 =13.6, p=0.0001), anxiety disorders (Chi2 =13.2, p=0.02), or sleep disorders (Chi2 =14.4, p=0.006), compared to the group of subjects without nitrous oxide use or the group of subjects without nitrous oxide use disorders.

**Conclusions:**

Nitrous oxide use and nitrous oxide use disorder were common among the health professions students included in the present study and correlated with daily alcohol or tobacco use.

**Disclosure of Interest:**

None Declared

